# Amplifying cancer treatment: advances in tumor immunotherapy and nanoparticle-based hyperthermia

**DOI:** 10.3389/fimmu.2023.1258786

**Published:** 2023-10-06

**Authors:** Yi Zhang, Zheng Li, Ying Huang, Bingwen Zou, Yong Xu

**Affiliations:** ^1^ Department of Radiation Oncology, Division of Thoracic Oncology, Cancer Center, West China Hospital, Sichuan University, Chengdu, China; ^2^ College of Management, Sichuan Agricultural University, Chengdu, China

**Keywords:** cancer, immunotherapy, nanoparticle, hyperthermia, photothermal therapy, magnetic hyperthermia therapy, tumor microenvironment

## Abstract

In the quest for cancer treatment modalities with greater effectiveness, the combination of tumor immunotherapy and nanoparticle-based hyperthermia has emerged as a promising frontier. The present article provides a comprehensive review of recent advances and cutting-edge research in this burgeoning field and examines how these two treatment strategies can be effectively integrated. Tumor immunotherapy, which harnesses the immune system to recognize and attack cancer cells, has shown considerable promise. Concurrently, nanoparticle-based hyperthermia, which utilizes nanotechnology to promote selective cell death by raising the temperature of tumor cells, has emerged as an innovative therapeutic approach. While both strategies have individually shown potential, combination of the two modalities may amplify anti-tumor responses, with improved outcomes and reduced side effects. Key studies illustrating the synergistic effects of these two approaches are highlighted, and current challenges and future prospects in the field are discussed. As we stand on the precipice of a new era in cancer treatment, this review underscores the importance of continued research and collaboration in bringing these innovative treatments from the bench to the bedside.

## 1 Introduction

**Figure 1:**
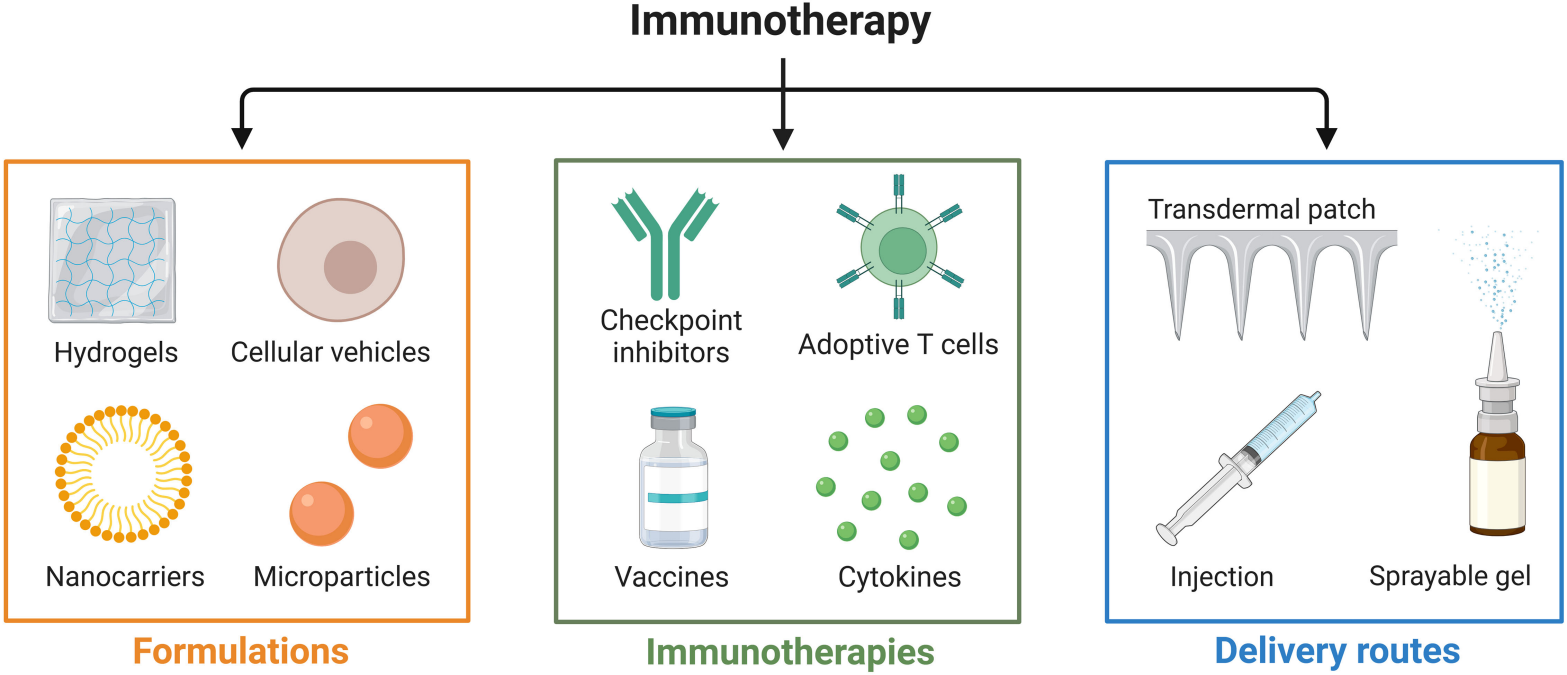
Recent advances in local delivery systems for cancer immunotherapy. Cancer immunotherapy methods, including immune checkpoint blockade (ICB), adoptive cellular therapy (ACT), and cancer vaccines.

Cancer is a global concern, with a substantial impact on human health. Tumor immunotherapy is capable of regulating the autoimmune system and restoring normal anti-tumor immune response, and has shown promising therapeutic potential in both *in situ* and metastatic tumors (1). Mainstream immunotherapies such as immune checkpoint blockade (ICB) (2), adoptive cellular therapy (ACT) (3), and cancer vaccines (4) have achieved good clinical results in the treatment of various tumors, as shown in **Figure 1**. Solid tumors, however, present barriers to drug delivery and are characterized by a complex tumor microenvironment (TME), resulting in ineffective infiltration of immune cells and low immune response rates, which greatly limits the clinical application of immunotherapy in solid tumors (5). As immunotherapy gradually shifts from monotherapy to multimodal combination therapy with immunotherapy at its core, oncologists have recognized that single immunotherapies are unable to cope with the complex, diverse, and dynamic immune escape mechanisms of tumors. Successful outcomes in the treatment of various cancers have been achieved by combining immunotherapy with radiotherapy, chemotherapy, and/or (gene-) targeted therapy; however, many of these regimens have resulted in a high degree of treated-related toxicity. Moreover, severe adverse effects and treatment resistance have been reported in patients receiving these combination therapies (6). In contrast, the combination of immunotherapy and hyperthermia produces synergistic effects by activating key systemic immune responses that enhance antitumor treatment (7). Serious adverse effects are minimal, and combination therapy has shown promising potential for clinical use (8). Thus, there is considerable value in exploring the combination of hyperthermia and immunotherapy in immunotherapy-centered cancer treatment with the goal of improving clinical outcomes in patients.

**Figure 2:**
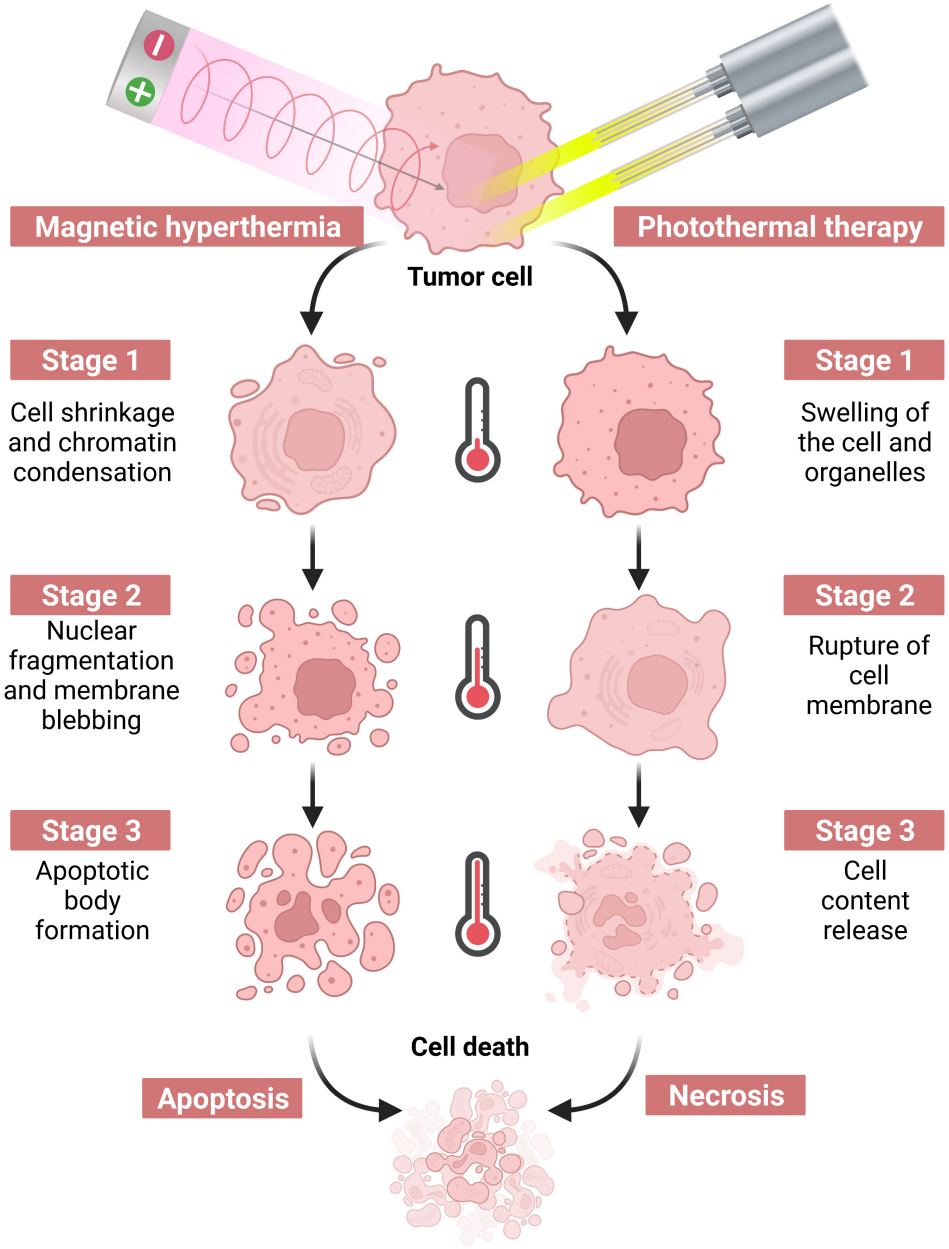
Hyperthermia therapy induced by a near-infrared laser or an alternating magnetic field causes tumor cell death via apoptosis or necrosis by increasing the temperature.

Tumor hyperthermia utilizes the bio-thermal effect of non-ionizing radiation to destroy tumor tissue or promote the death of tumor cells as a therapeutic technique, as shown in **Figure 2**. Specifically, Yonezawa et al. (9) showed that hyperthermia at 43°C induced apoptosis in malignant fibrous histiocytoma cells, whereas heat treatment at 44°C induced cell necrosis. A study by Harmon et al. (10) found that heating mouse mast cell tumor cells at 44°C for 30 minutes triggered apoptosis, whereas heating at 46°C for 30 minutes led to cell necrosis. Both apoptosis and necrosis can stimulate an immune response to some extent (11). Subsequent investigations by Geehan et al. (12) reported the first use of the immunomodulator interleukin (IL)-2 in combination with hyperthermia and found that combination therapy with these two treatment modalities reversed the immunosuppressive TME in mouse melanoma, enhanced the immune response against the tumor, and improved the response rate to immunotherapy. This encouraging finding led to a new wave of research on the combination of immunotherapy and hyperthermia for the treatment of malignant tumors. Conventional hyperthermia relies on energy sources such as light irradiation, electromagnetic waves, and mechanical waves; however, these approaches cannot be employed for the treatment of deep tumors, as they may damage normal tissue during the treatment process (13). Nanoparticle-based therapies have emerged as a revolutionary approach to cancer treatment by offering several advantages compared to traditional therapeutic methods. One of the most notable benefits of nanoparticle-based therapies is their ability to produce primarily local effects. Unlike chemotherapy and other systemic therapies, which can affect the entire body and lead to a range of undesirable side effects, nanoparticle-based treatments target the tumor directly. This localized approach ensures that any tissue damage is confined to the tumor and spares surrounding healthy tissues, including lymphoid tissue, outside the treatment field. Such therapeutic precision not only enhances the effectiveness of the treatment but also reduces the potential for adverse off-target effects. With recent advances in molecular biology and materials science, researchers are able to target tumor cells and tissues with precision at the microscopic level with heat-producing nanoparticles. The thermal effects of nanoparticles are stimulated by an applied energy field, which enables precision thermal strikes on cells and precise thermal therapeutic effects on tumors. By employing these techniques, the synergistic effects of hyperthermia and immunotherapy can be effectively achieved. Currently, nanoparticle-based photothermal therapy (PTT) and magnetic hyperthermia therapy (MHT) are the two principal techniques being investigated (8).

In the present review, we provide an overview of currently available hyperthermia therapies and explore the mechanisms of action underlying the activation of systemic immune responses with hyperthermia techniques. We also highlight recent advances in PTT and MHT in combination with immunotherapy for the treatment of tumors and provide a summary of the current outlook for these treatment modalities.

## 2 Nanoparticle-based hyperthermia and anti-tumor mechanisms

As an effective anti-cancer treatment, hyperthermia, which kills tumor cells or inhibits their growth by heating the malignant lesion, is predicated on differences in the temperature stress response between tumor tissue and healthy tissue. Normally, temperatures below 42°C have little to no cytocidal effect on cells, unless the exposure time is long (14). When cells are in a hypoxic or low pH state, however, the cytocidal effect produced by hyperthermia is more pronounced if the temperature is higher than 42°C (15). At temperatures below 43°C, apoptosis appears to be the primary pathway of cell death, whereas at higher temperatures, cell necrosis predominates. In addition, high temperatures can lead to double-strand breaks in DNA, which may result from the denaturation and dysfunction of heat repair proteins (e.g., DNA polymerase) or from the deposition of denatured proteins on nuclear chromatin structures, which prevents repair enzymes from reaching the site of damage (16). Hyperthermia-induced protein denaturation may also interfere with a variety of nuclear matrix-dependent functions (e.g., DNA replication, DNA transcription, messenger ribonucleic acid [mRNA] processing, and DNA repair). Thus, hyperthermia-induced cell death promotes antigen presentation or release through various mechanisms, in addition to increasing the signaling molecules that attract immune cells to the TME (17).

Based on the treatment modality, hyperthermia can be divided into three main categories: local, regional, and systemic (8). Whole-body, systemic hyperthermia is achieved with infrared light exposure or hot water baths, but is limited by the depth of penetration through the skin and is only used to treat skin or body-surface tumors (18). Regional and localized hyperthermia, on the other hand, delivers heat via electromagnetic or mechanical waves. Low-frequency electromagnetic waves can penetrate up to 15 cm into the body but are difficult to focus with precision. Higher frequencies are relatively easy to focus but cannot reach deeper tissues due to absorption by tissues. Therefore, electromagnetic wave-based hyperthermia is primarily limited to superficial tumors or the regional heating of deep tissues (13). In addition, at depths up to 20 cm, mechanical wave-dependent ultrasound generates heat with mechanical friction to achieve hyperthermia of deep tissues. This technique is currently approved by the US Food and Drug Administration for the treatment of cancer and can be used for ablation or mild hyperthermia. Disadvantages include a high absorption rate in bone tissue and the inability to penetrate tissues containing air, such as respiratory and gastrointestinal tissues (19). Emerging nanotechnology is driving advances in thermal therapy that effectively address these limitations.

PTT provides local thermal therapy to tumors by converting light energy into heat energy through photothermal converters under irradiation from an external light source, typically near-infrared light (700 ~ 900 nm range) (20). The conversion efficiency in the photothermal conversion process depends primarily on the material, shape, and surface properties of the photothermal conversion agent, among which gold nanorods and hollow gold nanoshells are the most commonly used (21). PTT limits the damage to adjacent healthy tissues by precisely controlling key parameters, such as laser power density, light wavelength, and irradiation time. These characteristics reflect the technique’s advantages of easy control and low adverse effects (20). However, due to the use of near-infrared light sources, tissue penetration depth is often limited to less than 1 cm, which renders PTT more suitable for the treatment of superficial tumors (20, 21). MHT is a form of heat therapy that relies on magnetic nanoparticles to convert magnetic energy into heat in an alternating magnetic field (AMF) (22). Magnetic nanoparticles are typically 2-20 nm in size and are administered either intravenously as a magnetic fluid or injected directly into the tumor. Iron oxide nanoparticles with superparamagnetic properties are the material of choice for MHT (23). To prevent the nanoparticles from aggregating in magnetic fields and to maintain spatial stability, the surface of iron oxide nanoparticles is typically coated with various materials, including a combination of polymers (24), chitosan (25), or silica (26), among others. MHT relies on AMF to induce thermotherapy; thus, there is no limitation on penetration depth *in vivo*, and the magnetic nanoparticles also promote tumor targeting (23). Currently, MHT has been found to be an effective therapy for deep tumors, and has achieved satisfactory clinical results in the treatment of brain, prostate, and esophageal cancers (22). Overall, nanoparticle-based PTT and MHT have considerable advantages over conventional hyperthermic therapy methods, including non-invasive, spatial, and temporal controllability, good targeting capability, and reduced adverse effects on healthy tissues.

## 3 Mechanism of action of hyperthermia in the activation of a systemic immune response

**Figure 3:**
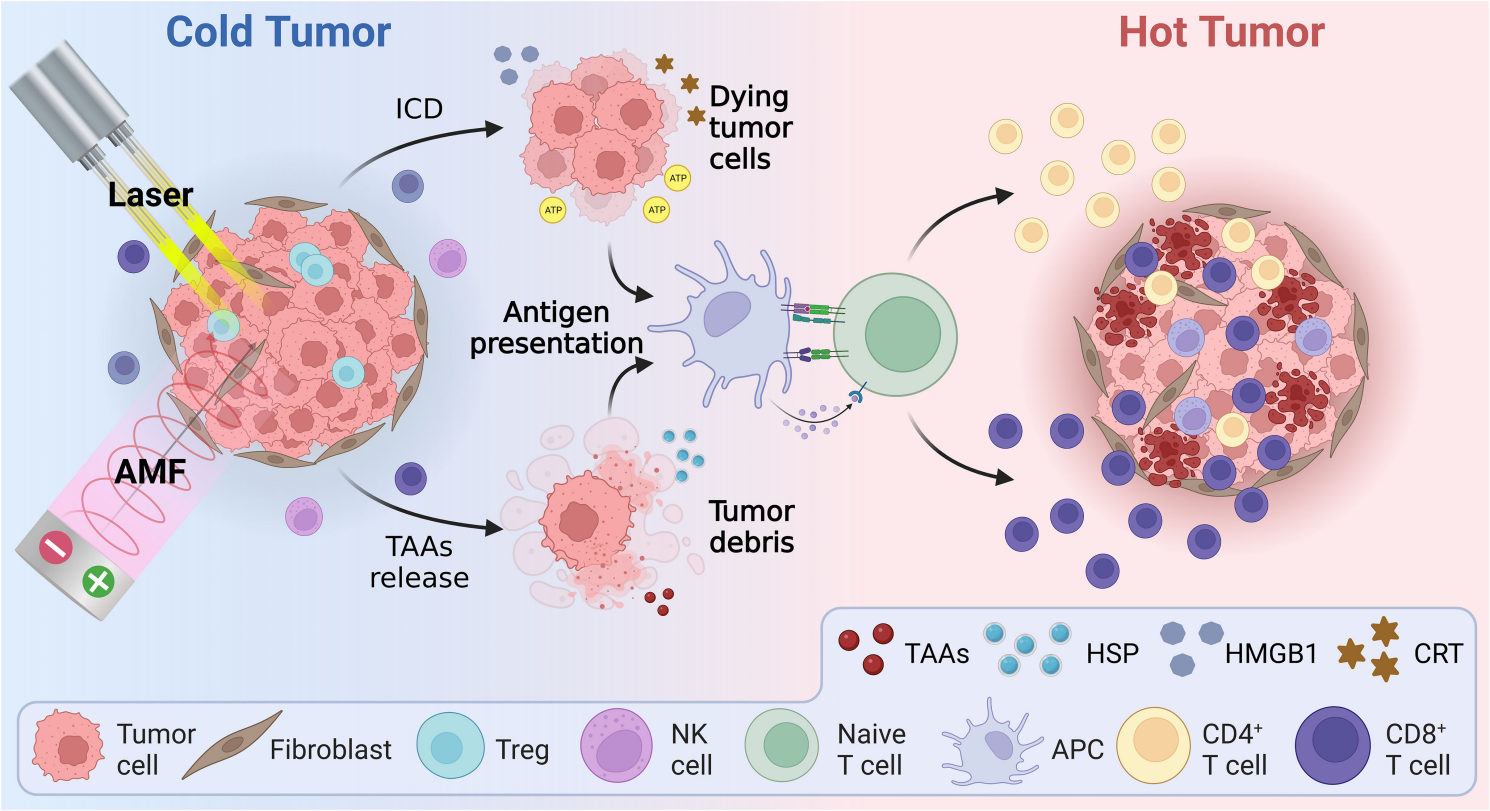
Schematic illustration of the mechanism of hyperthermia therapy combined with immunotherapy, including immunogenic cell death and reversal of the immunosuppressive tumor microenvironment. AMF, Alternating magnetic field; APC, Antigen presenting cell; ATP, Adenosine triphosphate; CRT, Calreticulin; HMBG1, High mobility group B1; HSP, Heat shock protein; ICD, Immunogenic cell death; TAAs, Tumor-associated antigens.

Hyperthermia can augment the body’s immunity against tumors, including effects that not only destroy tumor cells directly, but also activate the immune system by direct or indirect means, as shown in **Figure 3**. The exact mechanisms underlying hyperthermia’s effects on antitumor immune response, however, are not fully understood. Many studies have attempted to elucidate these mechanisms from a range of perspectives.

### 3.1 Effects of hyperthermia on the immune microenvironment and tumor cells

#### 3.1.1 Hyperthermia stimulates tumor cells to produce heat shock proteins

Hyperthermia can trigger the heat stress response. Heat shock proteins (HSPs) are key regulators of the heat stress response and are upregulated during heat shock to act as signaling molecules and transcription factors, which in turn activate the immune system to regulate the immune profile of the TME. In the case of disruption to cellular homeostasis, intracellular HSPs maintain their own structure and function by promoting protein folding (27). In addition, HSP70 may act as a specific target against tumors by increasing the density of CD56bright/CD94+ on the cell surface membrane of natural killer (NK) cells (28, 29). Moreover, extracellular HSPs released from tumor cells are known as potent enhancers of tumor antigen presentation and for stimulating anti-tumor immune responses (30). Immunogenic cell death (ICD) is a type of cell death that provokes an immune response in the presence of dead cell-associated antigens. HSPs, particularly during a state of hyperthermia, play a role in the facilitation of ICD, which involves not only the release of tumor antigens but also the release of other molecular factors, such as ATP, calreticulin, and HMGB1, which further modulate the immune response (31). More specifically, HSPs play a key role in antigen recognition by dendritic cells (DCs) by transporting antigenic peptides bound to HSPs to major histocompatibility complex-1 (MHC-I) molecules, which subsequently trigger the activation of antigen-specific T cells (32). This process is closely associated with ICD and promotes the phagocytosis of dying tumor cells by DCs, leading to a more potent adaptive immune response against the tumor (33). In addition, HSP60 (a chaperone protein) is involved in antigen-dependent T cell activation, in addition to stimulating interferon-γ secretion and T cell activation (34). In conclusion, HSPs transport a variety of peptide substances intracellularly, and form HSP–antigen complexes that are recognized by cytotoxic T cells and trigger specific immune responses (35). The comprehensive role of HSPs in ICD, and the subsequent activation of the immune response, underscores the therapeutic potential of targeting these proteins in cancer treatment (36).

#### 3.1.2 Hyperthermia promotes the secretion of cytokines

Hyperthermia stimulates the secretion of pro-inflammatory factors, including IL-1, interferon-γ, and tumor necrosis factor (TNF)-α, which are capable of directly destroying tumor cells and activating antigen-presenting cells (APCs) to attract them to the tumor site (37). Serum cytokine analysis found that applying heat therapy at 41°C for 30 minutes triggered an increase in inflammatory chemokines within the tumor and enhanced T cell migration (38). Specifically, mild hyperthermia increased the expression of L-selectin, P-selectin, and intercellular adhesion molecule-1 in the vessel wall, in addition to stimulating the production of cytokines such as IL-1β, IL-6, IL-8, IL-10, and CCL22. It is worth noting that the process of ICD is also characterized by the release of cytokines, highlighting the importance of elucidating the association between hyperthermia, cytokine release, and ICD in the context of tumor immunity (39). These factors are active in multiple phases of the immune response and facilitate the infiltration of immune cells in the TME. For example, in the acute inflammatory response stage, cryothermia-induced IL-6 drives the maturation of DCs, the differentiation of CD4+ T cells, and the generation of Th1-type antitumor immune responses (40, 41). Moreover, hyperthermia induces the secretion of CXCL10 and IL-6 by M1-type macrophages, drives the differentiation of CD4+ T cells into CD4+ CTL, Th1, and Th2 subpopulations, and reduces the proportion of myeloid-derived suppressor cells (MDSCs) (42, 43). Hyperthermia also acts directly on lymphocytes via IL-6 signaling, stimulates the MEK1-ERK1/ERK2 signaling pathway, enhances L-selectin adhesion, and promotes intermolecular interactions between the actin-based cytoskeleton, α-actin, and the cytoplasmic tail of L-selectin, which in turn enhances the adhesion and migration of L-selectin-independent lymphocytes (44).

#### 3.1.3 Hyperthermia alters the immunogenicity of tumor cells

Most chemotherapeutic drugs and radiation treatments used today primarily lead to apoptosis-driven cancer cell death rather than primary necrosis (45). However, hyperthermia-induced treatment pushes tumor cells towards necrosis, exposing novel tumor-associated antigens, thereby stimulating the immune system to initiate further tumor cell apoptosis and secondary necrosis. This necrotic cell death is termed ICD, which releases damage-associated molecular patterns (DAMPs) known to enhance the immune response against tumor cells. Not only does ICD release heat-shock proteins (HSPs), but it also manifests through the exposure of calreticulin on the cell surface and the secretion of ATP and HMGB1, acting as powerful immunostimulatory signals (46). In the tumor microenvironment (TME), high extracellular ATP levels stymie tumor growth through P2X7 receptors, activating P2X7-NLRP3 inflammatory vesicles and exerting P2X7-mediated cytotoxicity on tumor and tumor vascular endothelial cells, which in turn stimulates anti-tumor immune responses (28). Hyperthermia enhances this by increasing cell membrane lipid fluidity, improving P2X7 activity, boosting tumor cell death, amplifying tumor immunogenicity, and assisting immune cell-mediated tumor cell destruction (28). Nevertheless, cancer, stromal, and immune cells secrete VEGF-D and VEGF-C, promoting lymphatic vessel development in the TME, thus encouraging metastatic tumor proliferation in remote organs (47). Hyperthermia, on the other hand, suppresses VEGF-C/D secretion, differentiates monocytes into M1-type macrophages, and fosters inflammatory response factor formation, shaping a conducive environment for NK cells and cytotoxic T cells (48). The connection between dying cancer cells and immune cells critically determines cancer treatment efficacy, with ICD being triggered by adaptive immunity upon encountering stimuli like antigens, leading to enduring anti-tumor impacts (49). The antigenicity of tumor cells is usually minimal. However, with additional immune adjuvants, inflammatory agents, and chemokines, antigen-presenting cells (APCs) in the TME can now effectively recruit and activate T cells, inducing ICD. Among the known types of programmed cell deaths that can prompt ICD is pyroptosis (50). DAMPs, which include molecules like CRT, HSPs70, HSPs90, HMGB1, ATP, membrane-associated protein A1, and mitochondrial DNA, play a pivotal role in this (51). Pyroptosis, distinct from apoptosis and necrosis, is managed by inflammasomes and caspase-1/4/11 found in immune cells, essential for eradicating intracellular bacteria (52). Recent studies even tie hyperthermia with the induction of pyroptosis in cancer cells. For instance, in 2021, Li and colleagues discerned that photodynamic therapy initiates pyroptosis in esophageal squamous epithelial cancer cells through the PKM2/caspase-8/caspase-3/GSDME pathway (53). Further studies by Lu and his team also recognized a novel photosensitizer inducing pyroptosis (54). Hyperthermia, while damaging tumor cells directly and bolstering other treatments, induces pyroptosis, leading to a robust inflammatory reaction, augmenting the body’s inherent tumor cell recognition and elimination capabilities. Moreover, pro-inflammatory cytokines released during pyroptosis could enhance immunotherapy, strengthening the anti-tumor immune response.

### 3.2 Effect of hyperthermia on key immune cells in the TME

**Figure 4:**
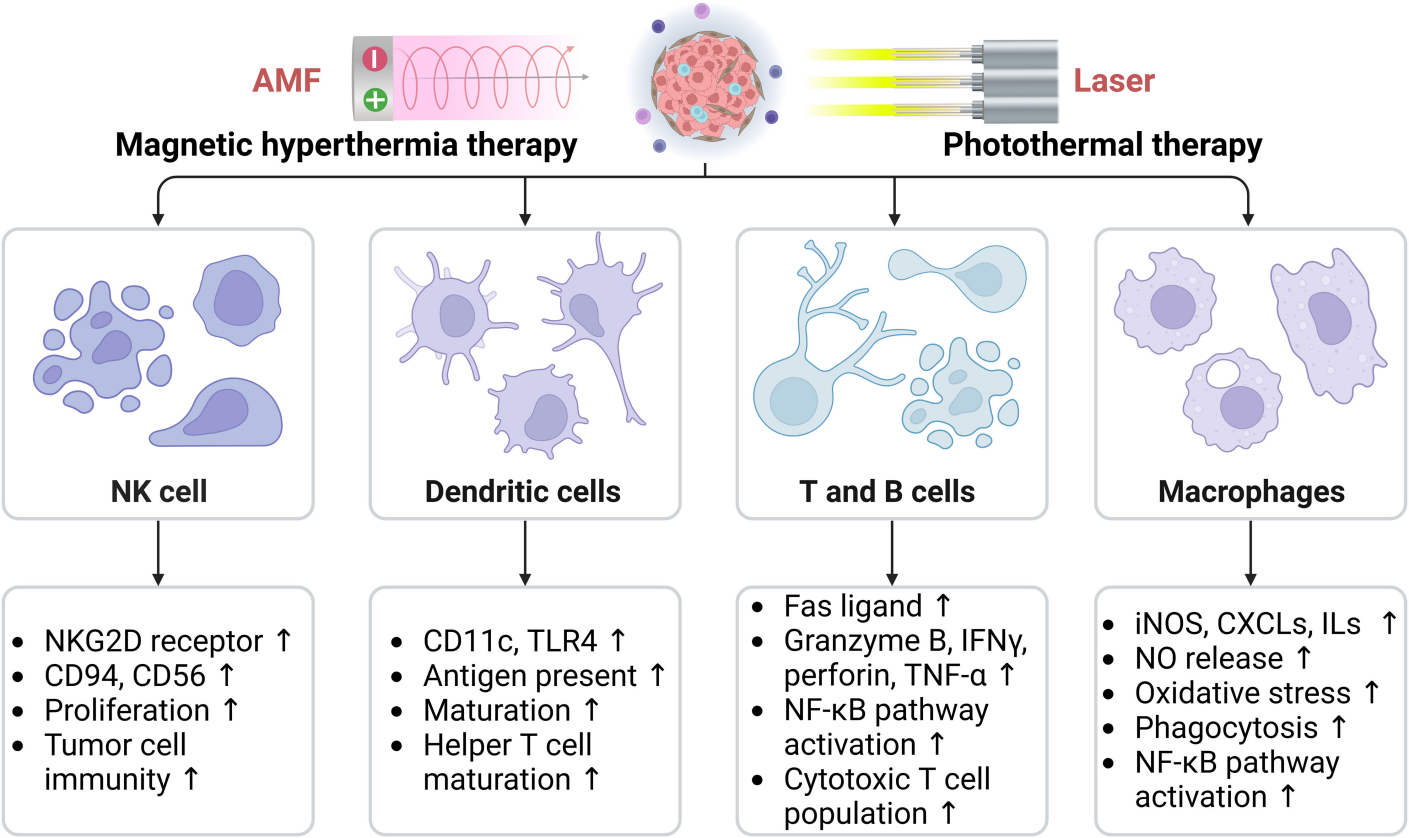
Effects of hyperthermia on key immune cells in the tumor microenvironment, including macrophages, dendritic cells, natural killer cells, T cells, and B cells. AMF, Alternating magnetic field; CXCLs, Chemokine (C-C motif) ligands; ILs, Interleukins; iNOS, Inducible nitric oxide (NO) synthase; NKG2D, Natural killer (NK) lectin-like receptor gene 2D; TLR, Toll-like receptor; TNF-α, Tumor necrosis factor-α. The arrows indicate the upregulation effect induced by hyperthermia.

Hyperthermia enhances the infiltration and function of macrophages, DCs, NK cells, T cells, and B cells, thereby activating the immune system and regulating the immune status of the TME (55). Herein, we have summarized the effects of hyperthermia on key immune cells located in the TME, including macrophages, dendritic cells, NK cells, T cells, and B cells (**Figure 4**).

#### 3.2.1 Hyperthermia and NK cells

NK cells, a type of lymphocyte of the innate immune system, are primarily responsible for the early recognition and attack of allogeneic and autologous transformed cells (56). Zanker et al. first investigated the effect of hyperthermia on NK cells and found that in patients with Ewing’s sarcoma, systemic hyperthermia at 41.8°C enhanced the cytotoxicity of NK cells (57). In addition, hyperthermia upregulated the expression of NKp30, the natural cytotoxic receptor of NK cells, and promoted the production of inflammatory cytokines IL-2 and IL-12, which rapidly activate NK cells. Activated NK cells significantly enhance the antitumor activity of NK cells by secreting IFN-α to promote Th1-type cell polarization and induce tumor-specific cytotoxic T cell activation (58). Notably, hyperthermia may have both enhancing and inhibiting effects on the tumor-cell killing activity of NK cells when the temperature is greater than 40°C (59).

The increased cytotoxicity of NK cells is dependent on the aggregation of natural killer lectin-like receptor gene 2D (NKG2D) surface receptors with ligands on tumor cells. High temperature-induced HSP70 promotes the expression of NKG2D ligands in a variety of cancer cell types, including colon, lung, and skin cancers (60), as well as the expression of NKG2D, CD94, and CD56 on NK cell membranes, thereby enhancing NK cell proliferation and activity (61). Moreover, HSP90, HSP72, and HSF-1 also enhance NK cell activity (62, 63). The killing capacity of NK cells is highest when both NK cell and tumor cell targets are activated (64). To date, the nature of heat-induced changes to NK cells that result in altered cytotoxicity have not been elucidated. However, according to Milani et al., this phenomenon is independent of the MHC class I/peptide complex (65).

During cytotoxic activity by NK cells enhanced by IL-2, the activation receptor NKG2D aggregates at the target cell contact site when pairs are formed between NK cells and target cells (66). Ostberg et al. studied the effect of mild heat stress on the localization of NKG2D on the surface of NK cells and found no change in the expression of NKG2D on the NK cell surface; however, a significant transient increase in NKG2D aggregation on the surface was observed. Moreover, cross-linking of NKG2D receptors with anti-NKG2D antibodies, or the IL-2 activation of NK cells, resulted in a similar aggregation effect (67). The activation status of NK cells may affect their sensitivity to heat stress, however, as IL-2 activated NK cells did not exhibit heat-enhanced cytotoxicity at 39.5°C (68). The aggregation of the activation receptor NKG2D may be related to the activation state of NK cells, which is enhanced by IL-2 activation; however, the temperature of the heat treatment cannot be further increased above 40°C. Therefore, high temperature does not appear to adversely affect IL-2 activated NK cells (69). In addition, higher heat resistance was observed in polycytidylic acid-activated NK cells compared to untreated NK cells at temperatures above 40°C (70). Therefore, depending on the state of NK cells and the various activation pathways, these cells may have different sensitivities to heat stress.

In conclusion, heat therapy enhances the immune effects of NK cells by activating them and improving their recognition of tumor cells. However, it is important to note that while hypothermia below a certain temperature threshold can be beneficial to NK cells, temperatures above that threshold may have detrimental effects.

#### 3.2.2 Hyperthermia and T cells

T cells are responsible for the elimination of mutant cells, bacteria, and viruses. Moreover, T cells produce antigen-specific cytotoxicity, effectively remove target cells, and are a key component of the antitumor immune response (71). Based on the type of cell surface receptors, T cells can be divided into CD3+/CD4+ (helper T cells) and CD3+/CD8+ (cytotoxic T cells). CD4+ T cell immunity is essential for the initiation of effector cytotoxic T cells (CTL), memory CTL development and PD-1/PD-L1 blockade (72, 73). CD4+ T cells can differentiate into a variety of helper T cells (Th) and regulatory T cells (Tregs) in different tumor environments. Th1 cells and the chemokines they produce exert antitumor activity by inhibiting neointima formation and promoting the recruitment of tumor-killing immune cells. FoxP3+ Tregs in the tumor impede effective anti-cancer immune responses and diminish the effect of PD-1/PD-L1 monoclonal antibodies; in contrast, Th17 cells in this context act as promoters of tumor growth (74).

Hyperthermia reconstitutes the TME to enable an effective response against PD-1/PD-L1 monoclonal antibodies by inducing CD4+ T cells to differentiate to Th1 and converting Treg cells to Th17 cells (75). In addition, hyperthermia can attract effector T cells to the TME by promoting the release of chemokines and breaking the vascular barrier, which promotes T cell infiltration (76). Moreover, whole-body heat baths have been found to decrease the total number of peripheral blood B and T cells, while CD8+ T cells were significantly increased, a finding that suggests hyperthermia enhances cellular immune function (77). An *in vivo* study found that exposure to moderate temperature hyperthermia greatly increased the translocation of L-selectin and α4β7 integrin-dependent lymphocytes to secondary lymphoid tissues (78), which led to an increased number of T cells in lymph nodes several hours after systemic hyperthermia. This observation suggests that heat therapy at appropriate temperatures stimulates T cell proliferation and recruitment. In addition, the response of T cells to heat therapy depends on the nature of the TME and its complex regulatory mechanisms. *In vivo* heat temperature can induce CD4+ T cell responses via Th2, which may be further regulated by the presence and concentration of IL-12 in the TME (79). In patients treated with systemic heat therapy, the decrease in T cells expressing α4β7 integrins was much smaller than the number of peripheral CD5-expressing T cells. Moreover, the effects lasted only a short time (80), suggesting that heat therapy inhibits the binding of T cells to integrin cell adhesion molecule (CAM)-1 and facilitates the recruitment of T cells into the TME, with a smaller effect on mature T cell function. Hyperthermia has also been shown to induce the expression of granzyme B, perforin, and interferon (IFN)γ, thereby increasing the cytotoxic activity of CTL (81). In addition, heat therapy induces the differentiation of CD8+ T cells into memory stem T cells (40).

#### 3.2.3 Hyperthermia and B cells

B cells produce antibodies and are a key component of humoral immunity (82). In addition, B cells play an active role in antitumor immune responses; for example, B cells can activate DCs or provide antigens for the activation and replication of CD4+ and CD8+T cells (82, 83).

Hyperthermia induces HSF1 expression by activating extracellular signal-regulated kinases and nuclear factor-κB (NF-κB) signaling pathways, thereby enhancing B cell proliferation and activation, and inducing TLR9 expression, which in turn activates an immune response (84–86). A study by Tomiyama et al. (87) showed that hyperthermia increased the expression of MHC class II molecules (e.g., HLA-DR) on the surface of T and B cells, thereby promoting their activation. High-temperature-induced HSF-1 has also been found to promote B cell proliferation and activation. In addition, hyperthermia can promote B cell-mediated immune responses by activating extracellular signal-regulated protein kinase and NF-κB signaling pathways and inducing B cell expression of TLR9 (85).

#### 3.2.4 Hyperthermia and dendritic cells

DCs are innate immune cells that play a key role in antitumor immunity by establishing a link between the innate immune response and adaptive immunity through phagocytosis of antigens. DCs have multiple phenotypes that effectively activate the adaptive immune system and express a range of activating and inhibitory receptors (88). Upon exposure to “danger” or other activating signals, DCs mature and activate primary T or B cells in the lymph nodes. In addition, DCs are capable of producing large quantities of pro-inflammatory factors.

Hyperthermia can promote the proliferation, maturation, and antigen presentation of DCs, which in turn activate adaptive immune responses. It has been shown that hyperthermia induces the maturation of DCs via heat shock transcription factor 1 (HSF1), HSP70, and toll-like receptor (TLR)-in a dose-dependent manner (89). During heat shock, tumor cells secrete HSP70, which acts as a DC antigen and enhances immunity against tumor cells through TLR4 (90, 91). In addition, HSP90 enhances immunity against tumor cells by forming tumor antigen–antibody complexes with donor cells (92). In addition, heat treatment-induced immunogenic cell death (ICD) is one of the mechanisms that enhances the immune effect of DCs against specific cancer types (93). Heat treatment has also been shown to induce a decrease in DC mitochondrial respiratory activity and oxidative phosphorylation. In addition, it increases glycolysis and the production of reactive oxygen species (ROS), promotes DC metabolic reprogramming, and shifts DCs from a quiescent to an activated state (94). Thus, heat treatment may prepare the immune system for the subsequent involvement of innate and acquired immune responses by promoting metabolic reprogramming of immune cells.

#### 3.2.5 Hyperthermia and macrophages

Macrophages, essential components of the innate immune response, govern both the inception and cessation of inflammation (95). Notably, these cells are predominant in the TME, forming approximately 50% of its cellular makeup and are referred to as tumor-associated macrophages (TAM). TAM primarily bifurcate into M1 and M2 types. M1 macrophages, when activated *in vitro* by agents such as IFN-γ, LPS, and GM-CSF, secrete pro-inflammatory cytokines and chemokines, thus bolstering innate and adaptive immunity and fostering apoptosis (30, 96). Conversely, M2 macrophages, stimulated by IL-4 or M-CSF, release anti-inflammatory molecules that generate anti-inflammatory effects, aid tissue reconstruction, encourage tumor angiogenesis, and suppress M1 macrophage functions, thus inhibiting apoptosis (97, 98). Hyperthermia, research has illuminated, induces immune responses by leveraging heat shock proteins (HSPs) to activate macrophages, with HSP70 and HSP90 in particular aiding in macrophage activation, phagocytosis enhancement, and related processes (99). Additionally, hyperthermia encourages M1-type cells to activate specific systems, producing robust anti-microbial and anti-tumor responses (100, 101). Therapies using heat and specific nanomaterials induce M1-type polarization in macrophages, amplifying immune surveillance, thereby thwarting tumor relapses and metastasis (42, 102). Notably, M2-type TAMs in the TME predominantly exert immunosuppressive actions (103). Manipulating TAM, either by curtailing their recruitment or steering them towards M1 polarization, has emerged as a promising avenue in cancer treatment research, though further investigations are imperative. Concurrently, both hyperthermia and macrophages activate the cGAS-STING pathway in cells (104). This pathway, vital for sensing DNA and kindling immune defenses, operates when cGAS detects extranuclear DNA, leading to a cascade that prompts the immune system, ultimately releasing inflammatory molecules such as IL-6 and TNF (105).

### 3.3 Effects of hyperthermia on other components in the TME

**Figure 5:**
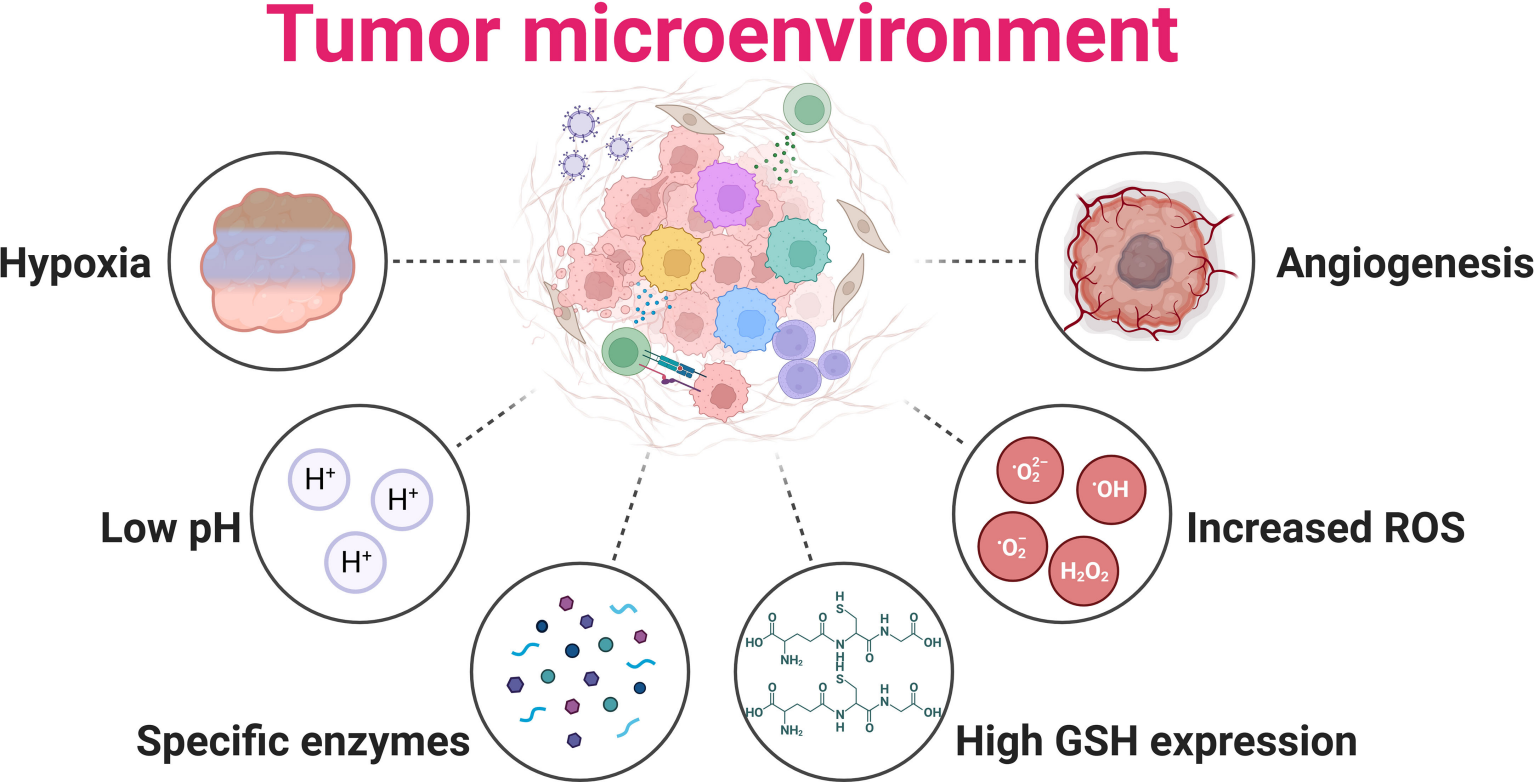
Tumor microenvironment hallmarks of solid tumor. Hypoxia: Tumor cells rapidly proliferate, often outpacing their blood supply, leading to insufficient oxygen availability within the tumor mass; Low pH: Hypoxia results from the proliferative metabolism, causing more lactate to accumulate due to glycolysis; Specific enzymes: Matrix metalloproteinases are expressed in response to various hormones and cytokines; High GSH expression: Glutathione (GSH) is upregulated in many tumors, providing an enhanced defense against oxidative stress and promoting tumor growth and survival; Increased ROS: High oxidative stress results from oncogene activation, antioncogene inactivation, mitochondria dysfunction, and aberrant metabolism; Angiogenesis: Tumors stimulate the growth of new blood vessels to supply themselves with oxygen and nutrients, supporting their rapid expansion and progression.

The TME is intricate, encompassing not only tumor cells but stromal cells, extracellular matrix, and various physicochemical factors. Unlike non-tumor tissues, the TME has distinct physiological attributes like altered pH, hypoxia, increased ROS, more specific enzymes, high GSH expression, and angiogenesis, as shown in **Figure 5** (106). Hyperthermia’s interaction with these elements can notably sway therapeutic outcomes. A hallmark of solid tumors is hypoxia due to the swift proliferation of tumor cells surpassing oxygen delivery from present blood vessels (107). This condition stabilizes hypoxia-inducible factors (HIFs), thereby fostering tumor advancement, metastasis, and therapy resistance (108). However, hyperthermia can potentially mitigate hypoxia by enhancing tumor oxygenation through increased blood flow, making tumor cells more receptive to treatments like radiation and chemotherapy (109, 110). Yet, extended hyperthermia may intensify hypoxia by inducing vascular closure (111). Tumors frequently have an acidic milieu due to augmented glycolytic metabolism, known as the Warburg effect (112, 113). This acidosis can shield tumors from treatments and boost their invasive tendencies (114). However, hyperthermia might counteract this by adjusting tumor pH, possibly amplifying the efficiency of combined therapies (115–117). Another facet is the elevated ROS levels in tumors, including H2O2, stemming from heightened metabolic activities and mitochondrial dysfunction (118). Hyperthermia can accentuate ROS in tumor cells, augmenting oxidative stress and potential cellular harm (119, 120). The synergy of intrinsic H2O2 and hyperthermia-induced ROS might intensify oxidative impairment, rendering tumor cells more prone to cell death (7, 121), though tumor cells could also bolster antioxidant defenses, which might diminish hyperthermia’s advantages. Finally, GSH, a pivotal cellular antioxidant, counteracts ROS and upholds cellular redox stability (122). Tumors often boost GSH production as a defense against heightened ROS (123), potentially conferring resistance to therapies by neutralizing drug-induced ROS and mending DNA damage (124). Hyperthermia’s impact on GSH in tumors is two-fold: while it might deplete GSH, making cells more susceptible to oxidative stress (125, 126), tumors might also bolster GSH production in retaliation to hyperthermia-induced stress, potentially diminishing cancer treatment effectiveness (127–129). Combining GSH level modulation strategies might thus amplify the therapeutic potential of hyperthermia in GSH-rich tumors.

## 4 Photothermal therapy combined with immunotherapy

PTT is a novel method of noninvasive tumor treatment that employs a photothermal agent (PTA) to kill tumor cells by converting light energy into heat energy under the irradiation of an external light source, such as near-infrared (NIR) light (130). As a non-invasive treatment, its advantages include a minimal side effects, high targeting accuracy, and repeatable treatment (131). Nanoparticle-based PTT is an emerging therapeutic approach that relies on the photothermal conversion ability of nanomaterials, and representative studies are shown in **Table 1**.

**Table 1:** NP-mediated PTT synergistic immunotherapy studies for the treatment of cancer.

Nanoparticles	Immunotherapy	Tumor type	Subjects	Ref
PANI-GCS	IA (R848)	Colorectal cancer	CT26, Balb/c mice	(25)
BP-BPEI-PEG	IA (CpG)	Breast tumor	4T1, Balb/c mice	(132)
PEG-PLGA-GRGDS-PFP	ICI (Anti-PD-1)	Melanoma	B16F10, C57B6 mice	(24)
GNS	ICI (Anti-PD-L1)	Glioma	CT-2A, C57BL/6 mice	(133)
PEG-SWNTs	ICI (Anti-CTLA-4)	Breast cancer	4T1, BALB/c mice	(134)
PLGA-ICG-R837	IA (R837) + IC I (Anti-CTLA-4)	Breast cancer, Colorectal cancer	4T1, CT26, BALB/c mice	(135)
GNs	ACT (TCR-T)	Melanoma	B16-F10, C57BL/6J mice	(136)
PLGA-ICG	ACT (CAR-T)	Melanoma	WM115, NSG mice	(137)
CINPs	Macrophage Repolarization (Antitumor M1-like phenotype)	Colorectal cancer	CT26, BALB/c mice	(138)
OVA-ICG	Cancer vaccine (OVA)	Melanoma	B16, C57BL/6 mice	(139)
HA-OVA-AuNPs	Cancer vaccine (OVA)	Lymphoma	EG7, C57BL/6 mice	(140)
AuNPs	Cancer vaccine (Tumor-derived vesicle)	Breast cancer	4T1, C57BL/6 mice	(141)

ACT, Adoptive cellular therapy; AuNPs, Gold nanoparticle; BP, Black phosphorus; BPEI, Branched polyethylenimine; CAR-T, Chimeric antigen receptor-modified T; CINPs, Cuttlefish ink nanoparticles; CpG, 5′- TCC ATG ACG TTC CTG ACG TT-3′; CTLA4, Cytotoxic T-lymphocyte antigen-4; GCS, Glycol-chitosan; GNs, Gold nano shells; GRGDS, Gly-Arg-Gly-Asp-Ser; HA, Hyaluronic acid; IA, Immune adjuvant; ICI, Immune checkpoint inhibitor; NGS, Nod scid gamma; NP, Nanoparticle; OVA, Ovalbumin; PANI, Polyaniline; PD-1, Programmed death 1; PD-L1, Programmed death-ligand 1; PEG, Polyethylene glycol; PFP, Perfluoropentane; PGNS, Plasmonic gold nanostars; PLGA, Poly(lactic-co-glycolic acid); PTT, Photothermal therapy; R837, Imiquimod; R848, Resiquimod; Ref, References; SWNTs, Single-walled nanotubes; TCR-T, T cell receptor-engineered T cell.

### 4.1 Photothermal therapy combined with immune checkpoint inhibitor therapy

Immune checkpoint inhibitor (ICI) therapy, also known as immune checkpoint blockers or blockade (ICB) is an antibody-dependent immune checkpoint suppression strategy that resists tumor immune escape by interrupting suppressive immune signaling pathways and activates the immune system to attack cancer cells (2). In clinical practice, common forms of ICI therapy include antibodies against programmed death receptor 1 (PD-1), its ligand PD-L1, and cytotoxic T lymphocyte-associated antigen 4 (CTLA-4) (1). Although ICIs)have made significant progress in recent years and are considered to be a highly promising area of investigation, their overall effectiveness in clinical applications, to date, ranges from 15% to 60% (142). Moreover, tumor cells can develop a drug-resistant TME through multiple mechanisms (143). In addition to the TME, tumor gene mutations, host immunity, and intestinal microecology may all have inhibitory effects on tumor immunity. As ICIs primarily target specific pathways, their efficacy may be limited. In this regard, it is critical to explore ways of targeting drug-resistant TMEs and to enhance tumor attack by pathways other than ICIs. Thermotherapy has shown great potential in this area; for example, a study by Shi et al. (144) found a significant increase in the ratio of CD+8 T/Tregs at mock metastatic sites, following ablative treatment of primary tumor foci, and a greatly enhanced capacity by tumor-infiltrating lymphocytes (TILs) to secrete IFN-γ and TNF-α in mouse models of colon cancer and melanoma. In addition, the combination of heat therapy with ICIs significantly controlled tumor volume and improved survival. Han et al. (145) verified the control of metastases following thermal ablation of the primary foci in a mouse experiment conducted with a combination of high-intensity focused ultrasound (HIFU) and anti-CTLA-4 adjuvant therapy. Consistent with the data reported by Shi et al. (144), Han and co-workers observed a decrease in the number of Tregs and MDSCs and found that HIFU increased the uptake of tumor-specific antigen (TSA) by DCs. The data showed that cytotoxic T lymphocytes (CTLs) at the metastatic site increased to 17.31%, while the percentage of Tregs decreased to 31.67%, which was much lower than the 54.05% in the untreated state, resulting in a 5-fold increase in the CD+8 T/Tregs ratio. Compared with ablation alone, the distant metastases in the heparin-induced thrombocytopenia (HIT) mice group were significantly regressed, with no significant adverse effects.

To address the problem of low overall objective response rates and high individual adverse reaction rates, several research teams have developed a combined nanoparticle-based thermal therapy and ICB treatment strategy and have made significant progress in preclinical trials. For example, Zhang et al. (24) found that the combination of photothermal therapy (PTT) based on PEG-polylactic acid-hydroxyacetic acid copolymer (PLGA) and iron oxide nanoparticles with anti-PD-1 exhibited substantial CD8+ T cell infiltration, almost complete inhibition of tumor growth, and significantly improved survival in mice compared to the control and anti-PD-1 groups alone. Furthermore, in addition to anti-PD-1 antibodies, the combination of nanoparticle-based PTT with anti-PD-L1 antibodies and anti-CTLA-4 antibodies also showed significant anti-tumor effects. Liu et al. (133) reported that the combination of gold nanostar (GNS)-based PTT with anti-PD-L1 antibodies showed considerable antitumor activity. The results of the study found that PTT of GNS combined with anti-PD-L1 antibody was significantly more effective in inhibiting tumor growth compared to anti-PD-L1 treatment alone. When reactivated in the tumor-free surviving mice, most mice exhibited durable immune memory and successfully resisted tumor reactivation. Wang et al. (134) found that single-walled carbon nanotube (SWNT)-based PTT in combination with anti-CTLA-4 antibody stimulated a strong adaptive immune response and suppressed both primary and metastatic tumor foci. In two mouse models of tumor metastasis, Wang et al. (134) found that combination therapy slowed tumor growth and significantly increased survival in mice at 50 days post-load (57% in the combination group vs. 25% in the anti-CTLA-4 alone group), and reduced the number of lung metastases from an average of 30 to 1. It is important to note that a study by Chen et al. (146) found that sequential thermotherapy decreased the density of Tregs in the TME, whereas single thermotherapy did not. Some reports suggest that incomplete ablation may promote metastasis of the primary tumor, whereas expanding the ablation may cause sub-lethal damage to the surrounding normal tissues; therefore, the control of temperature and range is particularly critical in the combination of PTT and ICB (147).

The findings described above provide a positive reference for the application of PTT in combination with ICB in clinical practice; however, larger clinical trials to further expand the current information of thermal dose, adverse effects of the combination, and the applicable population are needed.

### 4.2 PTT in combination with small molecule agonists (immune adjuvants)

Some small-molecule agonists (immune adjuvants), such as Toll-like receptor (TLRs) agonists, can be used in combination with nanoparticle-based PTT. The TLR agonists are non-specific immune enhancers that promote the maturation of DCs and the secretion of cytokines by activating TLR-related signaling pathways and subsequent systemic adaptive immune responses (148). Resiquimod (R848) is a potent TLR-7/8 agonist, and cytosine-phosphate-guanine-oligodeoxynucleotides (CpG-ODNs) are potent agonists of TLR-9.

Although TLR agonists have shown great potential as anti-cancer agents, *in vivo* instability, poor biodistribution and systemic toxicity severely affect their therapeutic efficacy (5). Recent findings suggest that nanotechnology offers a solution to this dilemma. Chen et al. (25) attached R848 and the photosensitizer polyaniline to glycol chitosan to form self-assembled R848@NP. The combined effect of PTT induced by near-infrared light irradiation and R848@NP-mediated immunotherapy inhibited tumor growth and induced durable immune memory, which effectively prevented tumor recurrence and metastasis. Yu et al. (149) synthesized DNA photosensitizer nanospheres (iDP-NS) with the immune adjuvant CPG-ODN and 808 nm photosensitizer ICG (laser irradiation triggered photodynamic and photothermal responses) in combination with PD-L1 to exert simultaneous anti-tumor effects. Zhao et al. (132) attached CpG-ODNs via electrostatic bonds to polyethyleneglycol (PEG)-polyethyleneimine-modified black scale nanosheets, such that the synergistic effect of nanoparticle-mediated PTT and CpG-ODNs increased the number of CD4+ T and CD8+ T cells in tumors by more than 26-fold under laser irradiation and significantly reduced the proportion of Tregs. In addition, serum levels of the anti-cancer cytokines IL-2, TNF- and interferon-y (IFN-y) were significantly increased, effectively eradicating the primary and metastatic tumor foci.

Immunoadjuvants are effective in enhancing the immunogenicity of tumor antigens and activating adaptive immune responses, while immune checkpoint blockade (ICB) impedes immunosuppressive activity by temporarily inhibiting the “molecular brakes” of the immune system (2). Thermotherapy stimulates a systemic antitumor immune response by inducing immune-related cell death (ICD) and reversing the immunosuppressive TME (7). Therefore, the combination of nanoparticle-based thermotherapy with these two immunotherapeutic strategies can lead to a potent synergistic antitumor effect against tumors. Chen et al. (135) used poly(lactic acid)-poly(ketone body) (PLGA) to co-encapsulate the TLR7 agonist imiquimod (R837) and the photothermal agent indocyanine green (ICG) to form PLGA-ICG-R837 nanoparticles that are capable of triggering photothermal ablation of tumor primary foci when excited by near-infrared light. The PLGA-ICG-R837 nanoparticles, excited by near-infrared light, can trigger photothermal ablation of the primary tumor site and induce higher-level maturation of DCs and a higher concentration and longer-lasting secretion of pro-inflammatory factors. Following combination anti-CTLA4 treatment in multiple mouse tumor models, the immune response continued to attack the remaining tumor cells, and the inhibition of tumor metastatic behavior was observed. In addition, this combination treatment demonstrated a strong immune memory effect after 40 days, providing effective protection against re-initiation of attack by the tumor.

### 4.3 Combination of PTT and macrophage immunotherapy

Tumor-associated macrophages (TAMs) are the most abundant population of immune cells in the TME and are divided into two main types: classically activated macrophages (macrophages 1, M1) and alternatively activated macrophages (macrophages 2, M2). M1-type macrophages have a significant anti-tumor function, whereas M2 macrophages are capable of driving tumor development (150). Currently, therapeutic approaches that induce a shift from M2-type TAMs to the M1-type have emerged as a new strategy for cancer immunotherapy. Recent studies have found that novel NPs are capable of inducing the shift from M2-type TAMs to the M1-type.

Deng et al. (138) extracted a cuttlefish ink-based (CINP) nanoparticles from cuttlefish ink with significant anti-tumor effects. The results showed that CINP could effectively induce a series of antitumor immune responses, including phagocytosis of tumor cells, antigen presentation, and antitumor factor production, by activating the mitogen-activated protein kinase signaling pathway to induce the shift of TAMs from the M2 to the M1 type. Under 808 nm laser irradiation, CINP not only ablated tumor cells via the PTT effect, but also increased the proportion of M1-type macrophages and CD8+ T cells in tumor tissues, which inhibited the growth of primary tumor and lung metastases. In addition, Qian et al. (26) constructed a biodegradable carbon nanodot mesoporous silica nanoparticles that not only mediated an enhanced PTT effect, under laser irradiation to ablate tumor cells, but also induced a shift of TAMs from the M2 to the M1 type, stimulated the proliferation and activation of NK cells, and increased the secretion of corresponding cytokines (e.g., IFN-y and granzyme B). Overall, combination therapy of nanoparticle-based PTT combined with tumor-associated M1-type macrophages produced potent antitumor effects, which are particularly suitable for specific types of tumors with a high degree of macrophage infiltration.

### 4.4 Combination of PTT and cancer vaccine therapy

Cancer vaccines are designed to trigger an intrinsic immune response against tumors, in addition to an adaptive immune response with the administration of preventive or therapeutic vaccines containing tumor antigens (4). Nevertheless, this approach currently presents challenges in terms of significant clinical efficacy, primarily due to difficulties in drug delivery that limit the immunogenicity and effectiveness of vaccines (5). However, with the rapid advances in nanobiotechnology, cancer vaccine research has acquired new possibilities.

Pan et al. (139) mixed the model antigen ovalbumin (OVA) with the photothermal agent indocyanine green (ICG) and labeled it using fluorescein isothiocyanate (FITC). It was shown in a mouse model that the FITC-labeled OVA-ICG nanovaccine was effectively phagocytosed by DCs. Moreover, labeled DCs were observed in the lymph nodes of mice, suggesting that the anti-tumor immune response had been activated. Compared to PTT alone and vaccine-based immunotherapy, OVA-ICG nanovaccine therapy significantly increased the number of CD8+ T cells in tumors, which was found to not only significantly inhibit the growth of melanoma cells but also triggered an immune memory effect that almost completely eliminated re-emerging melanoma. Similar findings were confirmed in an EG7 lymphoma model in mice (140). In addition, tumor-derived vesicles can also act as antigens to activate systemic immune responses. Zhang et al. (141) developed an immune nanosystem (AuNP@ DC B16F10) made up of gold nanoparticles as the core and mixed vesicles of melanoma cell and dendritic cell origin as the shell. Following subcutaneous injection, the accumulated AuNP@ DC B16F10 generated heat via the PTT effect to induce tumor ablation, along with the release of tumor-associated antigens. Together with AuNP@ DC B16F10, these antigens synergistically drive dendritic cell maturation, cytokine secretion, and T cell activation. Results from *in vitro* experiments showed that this combination therapy almost eliminated B16F10 tumors, delayed the onset of distant metastases, and substantially reduced tumor recurrence compared to PTT and immunotherapy alone.

### 4.5 PTT in combination with adoptive cell therapy

Adoptive cell therapy (ACT) is an immunotherapy technique in which immunologically active cells are isolated from the patient, activated and expanded *in vitro*, and subsequently reinfused into the patient, including T cell receptor gene-modified T cells (TCR-T), chimeric antigen receptor gene-modified T cells (CAR-T), and DCs. Although ACT has been shown to be clinically successful in the treatment of hematologic malignancies, the immunosuppressive TME in solid tumors, where it is difficult for lymphocytic cells to effectively infiltrate tumor tissue, has greatly limited the effectiveness of ACT in solid tumor treatment. However, as previously mentioned, thermotherapy can modify the TME so that it is more amenable to an immune response, thereby providing a promising avenue for improving the efficacy of ACT.

The use of permuted pmel T cells is a classic TCR-T treatment that uses genetic engineering techniques to introduce a TCR gene capable of specifically recognizing glycoprotein 100, a melanoma antigenic epitope, into CD8+ T cells, followed by amplification, after which it is infused back into the body. This technique enables increased recognition of tumor-associated antigens (TAAs), which improves clinical efficacy (151). Bear et al. (136) demonstrated that nanoparticle-based PTT in combination with permuted pmel T cells enhanced the antitumor effects. They used hollow gold nanoshells to induce PTT, followed by transplantation of pmel T cells. Ultimately the investigators found that this combination therapy was not only effective in halting the recurrence of the primary tumor but also inhibited the growth of distant tumors. Similar to pmel T cells, CAR-T cells are genetically engineered to form chimeric proteins *in vitro* by coupling the antigen-binding regions of antibodies capable of targeting specific TAAs to the intracellular portion of CD3-ζ chains or FceRγ, which are subsequently introduced into T cells. This technique promotes the stable expression of chimeric antigens on the surface of T cell membranes for the precise targeting of tumors (152). Chen et al. (137) injected poly(lactic acid)-poly(glycerol acid) copolymer (PLGA)-indocyanine green nanoparticles (ICGNP) into melanoma tumors in a mouse model, and after 2 h of infrared irradiation, injected CAR-T cells that could specifically recognize chondroitin sulfate proteoglycan 4 (CSPG4). The investigators found that PTT treatment resulted in increased blood perfusion in tumor tissues and decreased de-sensitization and interstitial pressure from tumor entities. Moreover, the direct cytocidal effect of PTT on tumor cells resulted in the release of TAAs, which contributed to the infiltration of CAR-CSPG4+ T cells into melanoma tumors. Compared to CAR-T treatment alone, the release of IL-2 and IFN-γ was significantly increased in mice after 20 days of combined treatment. In addition, tumor growth was significantly inhibited *in vivo*, and tumors disappeared to a naked eye in 2 out of 6 mice. These results suggest that nanoparticle-based PTT can effectively overcome the physical and immune barriers of solid tumors and enhance the accumulation and efficacy of pericytes of CAR-CSPG4+ T cells in solid tumors.

## 5 Combination of magnetic hyperthermia and immunotherapy

Magnetic hyperthermia (MH) is a method of thermal therapy that works by using magnetic nanoparticles (MNPs) in a high frequency alternating magnetic field (AMF) to induce the apoptosis of tumor cells by converting magnetic energy into heat via the Néel-Brownian relaxation mechanism, thereby elevating the tissue temperature at the lesion site and inducing apoptosis in tumor cells (153). After the external magnetic field is withdrawn, MNPs can enter the tumor-related area through the blood circulation or be recognized by the phagocytic system in the liver, spleen, and lymph nodes and cleared from the organism. Compared with other thermal therapies, such as radiofrequency and microwave techniques, magnetothermal therapy has the advantages of a high degree of safety, fewer side effects, and the ability to heat the tumor sufficiently, while being influenced by biological tissues with little effect on the magnetic field strength. Magnetothermal therapy, therefore, provides a novel strategy for the treatment of malignant tumors and enables precise thermal therapy at the molecular level (154, 155). Representative studies of nanoparticle -based MHT are shown in **Table 2**.

**Table 2:** NP-mediated MHT synergistic immunotherapy studies for the treatment of cancer.

Nanoparticles	Immunotherapy	Tumor type	Subjects	Ref
IONs	IA (IL-2)	Lung cancer	Lewis, C57/BL6 mice	(156)
MCLs	IA (IL-2)	Melanoma	B16, C57BL/6 mice	(157)
PEG-FVIOs	ICI (Anti-PD-L1)	Breast cancer	4T1, Balb/c mice	(158)
PEG-FeNPs	IA (R848) + ICI (Anti-CTLA-4)	Breast cancer, colorectal cancer	4T1, CT26, Balb/C mice	(159)
MCLs	ACT (DCs)	Melanoma	B16, EL4, C57BL/6 mice	(160)

ACT, Adoptive cellular therapy; CTLA4, Cytotoxic T-lymphocyte antigen-4; DCs, Dendritic cells; FeNPs, Pure iron nanoparticles; FVIOs, Ferrimagnetic vortex-domain iron oxide nanoring; IA, Immune adjuvant; ICB, Immune checkpoint inhibitor; IL-2, interleukin-2; IONs, Iron oxide nanoparticles; MCLs, Magnetite cationic liposomes; MHT, Magnetic hyperthermia therapy; NP, Nanoparticle; PD-L1, Programmed death-ligand 1; R848, Resiquimod; Ref, References.

### 5.1 Combined magnetothermal therapy with immune checkpoint blockade

In an investigation by Pan et al. (161) of monodisperse CoFe2O4@MnFe2O4 nanoparticles in a mouse model of breast cancer, it was found that the nanoparticles were capable of releasing efficient, stable, and controlled thermal energy in an alternating magnetic field. The number of CD8+ T cells was significantly increased locally in the tumors of mice treated with thermotherapy combined with ICBs and this group of mice had the lowest mortality rate. In addition, the combination of magnetic heat therapy with ICBs resulted in the regression of solid tumors and prevented tumor metastasis. Liu et al. (158) investigated the antitumor effects of ferrimagnetic vortex-domain iron oxide nanoring (FVIO)-mediated magnetic heat therapy in combination with anti-programmed death protein ligand-1 (PD-L1) therapy in breast cancer. The investigators found that FVIO-mediated mild magnetic heat therapy induced calreticulin expression in 4T1 breast cancer cells, which further promoted phagocytosis of cancer cells by the immune system and efficient death of immunogenic cells, as well as polarization of macrophages. Moreover, this therapy increased the infiltration of CD8+ cytotoxic T cells in distant tumors, which was further increased from 55.4% to 64.5% with the combination of PD-L1 inhibitors. In addition, the combination therapy was superior to either FVIO-mediated magnetothermal therapy alone or anti-PD-L1 therapy alone in several mouse model experiments for the treatment of primary 4T1 tumors, prevention of lung metastases from 4T1 tumors, and inhibition of distant tumor growth. Furthermore, the treatment regimen did not cause significant pathological changes in major organs and had an acceptable safety profile. Thus, the strategy of combined magnetothermal therapy and anti-PD-L1 immunotherapy deserves further development and investigation due to its excellent antitumor effects, including the effective inhibition of tumor recurrence and metastasis.

### 5.2 MHT co-immunomodulators (immune adjuvants)

Interleukin is an immunomodulator that coordinates the activity of various types of immune cells. IL-2 regulates the growth and differentiation of T and B cells and has long been recognized as an immune adjuvant (162). Although IL-2 has demonstrated considerable potential in the treatment of metastatic cancers, its clinical application has been restricted due to several limitations. The first limitation concerns the bidirectional immune effect of IL-2, in that it promotes both effector and regulatory T cell (Treg) expansion. Moreover, due to the short half-life of IL-2 in serum, high doses are often required for administration, which may trigger toxic reactions in the affected organism (5). One study found that IL-2 might benefit from the immunostimulatory effects of thermotherapy. In a mouse model of lung cancer, investigators found that IL-2 combined with nanoparticle-mediated MHT treatment increased infiltration of CD4+ and CD8+ T cells, and that tumor growth inhibition was significantly higher with combination treatment (68.1%) compared to IL-2 (14.2%) or MHT alone (45.8%) (156). Similar results were obtained in a mouse melanoma model (157), in which the combination of PTT mediated by magnetite cationic liposomes, IL-2, and granulocyte macrophage colony-stimulating factor significantly enhanced the antitumor immune response, dramatically reduced tumor burden, and increased survival compared to monotherapy. These results suggest a beneficial synergistic relationship between nanoparticle-based MHT and immunomodulators, although further studies are needed to clarify the changes in Treg cells exposed to combination therapy, in addition to determining the appropriate IL-2 dose to mitigate the toxicity associated with high-dose IL-2 therapy.

The therapeutic strategy of nanoparticle-based MHT in combination with immune adjuvant and ICB has also been performed in preclinical trials. Chao et al. (159) found that the combination of pure iron nanoparticle-mediated MHT in alternating magnetic fields (AMF), local injection of nanoparticle, and systemic injection of anti-CTLA4 antibody inhibited tumor metastasis and increased the memory T cell ratio, thereby inducing a strong and long-lasting anti-tumor immune memory and effectively preventing tumor recurrence. In addition, Chang et al. (7) prepared bismuth selenide magnetic nanocages containing R848 immune adjuvant that, combined with anti-PD-L1 therapy, significantly enhanced the effect of immunotherapy alone and achieved effective treatment of the tumor.

### 5.3 Combination therapy of MHT with ACT

To date, nanoparticle-based therapeutic strategies for MHT in combination with ACT are also being developed in a variety of murine tumor models. Tanaka et al. (160) evaluated the efficacy of magnetothermal therapy based on magnetite cationic liposomes with pericytes against tumors in a mouse model of B16F10 melanoma and ELAT lymphoma. The results showed that tumor regression was complete in 60% of mice in the combination treatment group, whereas no tumor regression was observed in the mice treated with either heat therapy or DCs alone. In addition, CD8+ T lymphocyte and natural killer cell activities were significantly increased in the combination treatment group, and the mice with complete tumor regression were able to resist secondary attacks by tumor cells, demonstrating that nanoparticle-based magnetic thermotherapy combined with DC treatment can prolong tumor immunity, creating new possibilities for the use of eosinophilic lymphocytes for clinical applications in solid tumors.

## 6 Conclusion and outlook

Immunotherapy offers novel strategies for the treatment of a wide range of cancerous tumors; however, several clinical challenges remain, including low patient remission rates and dose-limiting toxicity. Nanomaterials offer a potentially innovative approach to precision thermotherapy due to their unique physicochemical properties. Nanotechnology-based drug carriers optimize the pharmacokinetic properties of conventional drugs and improve bioavailability, along with reduced toxicity and adverse effects. The synergistic therapeutic strategy of nano-based thermal therapy combined with immunotherapy addresses these factors. By inducing immune-related cell death in tumor cells, reversing the suppressive TME, and increasing the penetration and retention of nanoparticles in solid tumors, among other mechanisms, nanoparticle-based PTT and MHT are capable of triggering a systemic antitumor immune response. In synergy with multiple immunotherapies, nanoparticle PTT significantly enhances the immunogenicity of tumors and improves the clinical benefit of immunotherapies used alone (7, 163, 164). In addition, the use of magnetic targeting agents and functionalized modifications of nanoparticle formulations have enhanced tumor-specific heating. Moreover, the development of novel nanoparticle formulations and morphologies has improved thermal conversion efficiency and addresses the limitations of tumor uptake of nanoparticles (165). These technological advances not only remove considerable barriers to the widespread use of nanoparticle-based thermotherapy, but further enhance the antitumor benefits of both therapeutic modalities.

The future development of tumor immunotherapy combined with nanoparticle-based thermotherapy should ideally be based on advances in both thermotherapy and immunotherapy. Exploring new potential immunotherapeutic targets, developing new inhibitors, further defining biomarkers for immunotherapy, improving efficacy, and reducing toxicity and side effects remain the future goals of immunotherapy. In terms of thermal therapy, real-time temperature measurement, precise temperature control, and accuracy are the primary research objectives. Moreover, 3D non-invasive thermometry based on magnetic resonance (MR)-guided and computed tomography (CT) is rapidly developing. Currently, advanced infrared real-time thermometry is capable of detecting local energy distribution and providing 2D thermograms, which provide a better platform for thermal therapy quality control (166).

Looking ahead, four key issues to be addressed in this field are the following: 1) The biosafety of nanoparticle formulations requires systematic evaluation, either by increasing the number of toxicological studies in humans or by employing nanoparticles with good biodegradability to accelerate their excretion. The adverse effects of co-administration of nanoparticles and thermotherapy should also be an area of focus. 2) The targeting and stability of nanoparticle formulations *in vivo* needs to be improved; for example, by introducing targeting ligands, using external magnetic fields for precise magnetic targeting, or employing biomimetic cell membrane coatings to promote immune escape and homologous targeting (165). 3) The optimal thermal dose of nanoparticle-mediated thermotherapy in synergistic therapy needs to be established. In addition, temperature monitoring techniques need improvement, and dynamically monitoring the immune response at different thermal doses after synergizing different immunotherapies needs to achieve optimal efficacy. 4) Validation of the efficacy of nanoparticle-based thermal synergistic immunotherapy is currently limited to mouse models and needs to be gradually expanded to other animal models to accelerate its translation into clinical practice.

## Author contributions

YZ: writing – original draft, writing – review & editing. ZL: writing – original draft, writing – review & editing. YH: writing – original draft, writing – review & editing. BZ: conceptualization, funding acquisition, supervision, writing – review & editing. YX: conceptualization, funding acquisition, supervision, writing – review & editing.
